# Health promotion preparedness for health crises – a ‘must’ or ‘nice to have’? Case studies and global lessons learned from the COVID-19 pandemic

**DOI:** 10.1177/1757975921998639

**Published:** 2021-03-27

**Authors:** Diane Levin-Zamir, Kristine Sorensen, Tin Tin Su, Tetine Sentell, Gillian Rowlands, Melanie Messer, Andrew Pleasant, Luis Saboga Nunes, Shahar Lev-Ari, Orkan Okan

**Affiliations:** 1 Clalit, Tel Aviv, Israel; 2 School of Public Health, University of Haifa, Haifa, Israel; 3 Global Health Literacy Academy, Urmond, Netherlands; 4 Monash University, Bandar Sunway, Malaysia; 5 Office of Public Health Studies, University of Hawai‘i at Mānoa, Hawaii, United States; 6 Newcastle University, Newcastle upon Tyne, United Kingdom; 7 APOLLON Hochschule der Gesundheitswirtschaft (University of Applied Sciences), Bremen, Germany; 8 Health Literacy Media, United States; 9 University of Education Freiburg, Freiburg im Breisgau, Baden-Württemberg, Germany; 10 Tel Aviv University Sackler Faculty of Medicine, Tel Aviv, Israel; 11 Bielefeld University, Bielefeld, North Rhine-Westphalia, Germany

**Keywords:** health literacy, health-promoting schools, equity/social justice, health-promoting healthcare, vulnerable groups, migrant health, mental health literacy

## Abstract

The current COVID-19 pandemic has exposed missing links between health promotion and national/global health emergency policies. In response, health promotion initiatives were urgently developed and applied around the world. A selection of case studies from five countries, based on the Socio-Ecological Model of Health Promotion, exemplify ‘real-world’ action and challenges for health promotion intervention, research, and policy during the COVID-19 pandemic. Interventions range from a focus on individuals/families, organizations, communities and in healthcare, public health, education and media systems, health-promoting settings, and policy. Lessons learned highlight the need for emphasizing equity, trust, systems approach, and sustained action in future health promotion preparedness strategies. Challenges and opportunities are highlighted regarding the need for rapid response, clear communication based on health literacy, and collaboration across countries, disciplines, and health and education systems for meaningful solutions to global health crises.

## Introduction

The COVID-19 pandemic has exposed missing links between health promotion and national/global emergency policies. Health promotion, by enabling people to increase control over their health, is essential when health crises such as COVID-19 emerge. Throughout the course of the pandemic, the role of the public in mitigating the crisis has been emphasized, as health promotion needs emerged, including:

- maintaining healthy lifestyles during periods of lockdown/curfew (1)
- empowerment and self-care for all, while healthcare and educational systems rapidly shifted orientation to digital navigation, COVID-19 prevention and care (2)
- mental health challenges across the lifespan (3)
- coping with the infodemic (4).

Vulnerable populations were left behind due to lack of attention given by authorities to health literacy (5), language or cultural needs, and/or limited access to health promotion services and opportunities (6). These are not the sole factors leading to the known health inequalities in risks of contracting, and dying from, COVID-19; rather, they compound other structural inequalities in health, such as poor housing, residential density, and a higher likelihood of holding low-paid occupations requiring in-person contact (7). Without clear-cut contingency plans, health promotion action was not the initial focus in many countries. As the pandemic spreads, subsides and reappears, people have been challenged by uncertainty, and with the understanding that individual needs should service collective needs (8). Among the essential tools recognized for containing and mitigating COVID-19, accessible, trustworthy, understandable, culturally appropriate, and timely information addressing health literacy needs (9) is recognized as a condition for achieving positive health behavior change. As prevention and prediction are now key words that flood the public discourse, lack of health literacy erodes support for strong public engagement (10).

As health promotion is essential for overcoming the pandemic and changing health behavior (11), initiatives have been urgently developed and immediately applied globally during the outbreak. A selection of case studies from regions across the world are presented here to exemplify challenges and ‘real-world’ action for health promotion practice, research and policy as described by health promotion/health literacy experts during the COVID-19 pandemic. The aim was not to provide a comprehensive overview, but rather to illustrate different contexts, settings, national and local policies and varying guidelines aimed at reducing the risk of infection and/or transmission. Health promotion and health literacy are interdisciplinary (12) and international fields, reflected in the case studies chosen. The actions range from individual intervention through action focused on family, community, healthcare, public health and media systems, health promoting settings and policy. These cases and this special issue highlight a bright light emerging from the pandemic, which is that rapid response, communication, and collaboration across nations, disciplines, and perspectives can build successful solutions to an unprecedented global health crisis. Health promotion efforts are often constructively considered within the context of the socio-ecological model (SEM) (13). This model provides a framework that situates individual health outcomes, knowledge, and behaviors in the context of influence from *individual, interpersonal, organizational, community*, and *policy-level* factors (14). Each layer of influence on health behavior change may be exemplified thusly:

**Individual**: knowledge, attitudes, self-efficacy, health literacy skills, values, personal psycho/social/demographic attributes;
**Interpersonal**: people with close relationships to the individual: immediate family, relatives, close friends and co-workers, peer network;
**Organizations**: agencies, social institutions, public/private partnerships;
**Community**: schools, workplaces, neighborhoods, places of worship, community primary care;
**Policies**: national healthcare organizations, governmental ministries, mandates, laws.

**Figure 1.**
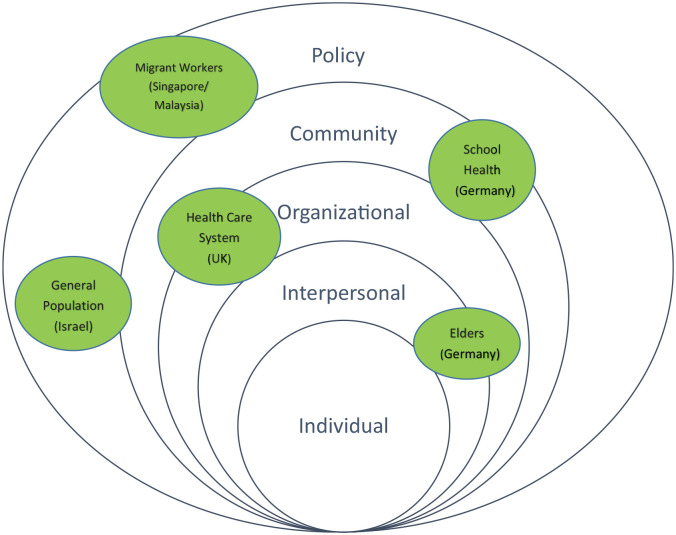
Cases in the context of the social-ecological model.

This model also offers opportunity to explore cross-level interventions in the socio-environmental context. Figure 1 provides a visual of these contexts based on the following cases presented. Our purpose is to showcase needs across the lifespan, best practices exemplified through the SEM, lessons learned and conclusions regarding the application of health promotion practice during the pandemic.

## Case 1: interpersonal focus — older people coping with COVID-19 (Germany)

### The needs

In March 2020, two months after the first reported case in Germany, nationwide public health measures were announced, including contact restrictions, closure of public facilities, and even stay-at-home orders issued by some federal states. The measures were intended to contain the epidemic, increase the capacity of the healthcare system to treat COVID-19, and protect vulnerable populations, in particular older people and people with pre-existing conditions (15). Yet, more specific action was needed to meet the needs of the elderly.

### The actions

Several laws were amended to enable the health sector to respond to epidemics (15). Early after the outbreak, federal health authorities implemented nationwide public health campaigns and promoted health-related media communication. Messages called for solidarity within society (e.g. ‘Together against Corona,’ promoting shopping for older neighbors), mutual support and adherence with official recommendations. While early in the pandemic, health information was general in nature, by April 2020, health authorities released more specific health information, tailored to the needs of older people (16).

Primary care providers introduced temporary measures such as telephone and video-based consultations, suspended mandatory check-ups and disease management programs, and allowed refunds of postal charges for medical prescriptions (17). Precautions to protect older people living at home included stay-at-home recommendations and limited family contact (e.g. restricting grandchildren’s visits). Older people living in nursing home facilities were strictly isolated, by banning visits, canceling group activities, and forbidding residents to leave the facility (15,18). Measures addressed residents, their relatives, service providers (e.g. hairdressers), and health professionals (18). Additionally, health authorities introduced specific health recommendations for long-term care (19).

Notably, temporary measures were applied to safeguard citizens’ economic situation, including protection against eviction for tenants, and to support nursing care and care by relatives.

### Lessons learned

Systematic public evaluation and robust data analysis are not available yet. At the federal state and local levels, evaluation and readjustments of measures and laws are ongoing. The Federal Public Health Institute presents daily situation reports (19), supported by the scientific community providing evidence, analysis of healthcare, and recommendations.

Adverse effects of social isolation and loneliness on older people’s health, and barriers to healthcare for people with chronic conditions are severe and have been documented (18,20). Crisis hotlines for immediate counseling reported twice the number of calls in April (21). The main concerns of the callers were loneliness, psychological stress, family crises, and fear of inadequate care. Once a considerable decrease in doctor visits was noted, people were urged not to delay or postpone critical visits. The importance of relatives, home care services, and nursing homes for prevention and containing the spread of infection, and for ensuring proper support and care of older people, became evident. Furthermore, earlier support measures were adapted and strict isolation measures in nursing homes were relaxed. Early in the pandemic, measures were dominantly aimed at increasing patient safety. Yet, as the pandemic progresses, measures need to shift toward specific patient-centered health promotion, community health support, and appropriate health information interventions based on the health literacy needs of older people. New measures should also help to improve living and working conditions, enable social participation and quality-of-life for older people, and empower all involved to better cope with the changing pandemic situations.

## Case 2: organizational focus — mental health and the view from primary care (UK)

### The needs

Between the first COVID-19 case in the United Kingdom in January 2020, and when the country went into a strict lockdown in March, anxiety increased about the possibility of severe illness and death. There was deep concern about overwhelming health services and not having sufficient facilities to care for people if they became ill. More than two-thirds of adults reported feeling worried about the effect of COVID-19, with over half reporting stress and anxiety (22).

Health services increasingly focused on providing intensive and critical care and on diagnosing COVID-19. Hospital and other specialist services were scaled back or even stopped. Access to mental health services for people with pre-existing conditions was 25% lower than before the pandemic (23), and likely even lower for people without pre-existing mental conditions. Some health services, such as General Practice (GP) remained fully available, albeit with consultations largely virtually or by phone. However, because the public assumed that GPs were not available, many did not access the service. This resulted in a stressed and anxious population without access to mental health advice and support, placing especially at risk those with low access to digital information, such as the socioeconomically deprived.

### The actions

The importance of supporting mental health during the pandemic was initially championed by mental health charities such as the Mental Health Foundation (24), MIND (25), and the Royal Family; the Duke of Cambridge, in particular, has a longstanding interest in mental health (26). The National Health Service made mental health advice available online (27). The public was reassured that non-COVID health services such as GP were still available for advice and support for conditions, including mental health. Efforts were made to ensure information was easy to access, understand and use, although inequality in access to online information persisted.

### Lessons learned

Weekly national evaluation of the epidemic’s social impact continues. The latest available findings at the time of writing show that the proportion of the population worried about the effect of COVID-19 on their life remains high at 70%, but fewer people are feeling stressed and anxious, as lockdown eases and life starts to return to a ‘new normal’ (28). Yet, the proportion of people feeling stressed and anxious remains much higher than before the pandemic (29). The pandemic exacerbated existing mental health inequalities; populations whose mental health is at greatest risk include those with existing mental health problems, people with long-term physical conditions, women and children experiencing violence and abuse, and Black, Asian, and minority ethnic (BAME) communities (30).

The pandemic also increased opportunities to discuss mental health, and hence increased mental health literacy. More than two-thirds of the population feel able to express feeling worried about the effect of the pandemic, with more than half describing stress and depression. This demonstrates a shift from previous stigmas creating barriers to admitting, discussing, and seeking care for mental health problems. Recognition of the need to promote mental health in parallel with physical health (6) has increased. Efforts should be made to maintain this momentum in both the short and the long term. Increased mental health resilience will become especially important in the ‘second wave’ of the pandemic.

Mental health resources must target those most at risk, focusing on building skills among the least advantaged, including applying health literacy approaches. The current monitoring systems must continue to ensure that mental health improves and inequalities are reduced. Healthcare organizations working in partnership especially with mental health-focused advocacy groups can continue to share the message from their organizational perspectives to change community and interpersonal norms and understanding about mental healthcare access and stigma.

## Case 3: community focus — school health promotion and COVID-19 (Germany)

### The needs

Germany’s first reported case of COVID-19 was on 27 January 2020 (15). In Germany, there are about 13.6 million children under 18 years of age. Children and schools were long neglected by the public health system (31,32). Schools in Germany are critical settings for socialization and health promotion for children (33), especially those vulnerable and disadvantaged, who may experience adverse health consequences when confronted with abrupt changes. In particular, social and educational development in the children involved may suffer long-lasting effects. The turmoil caused by the COVID-19 pandemic, potentially damaging to children, must be carefully addressed during and in the aftermath of this crisis (31,32).

### The actions

Only in mid-March 2020 did the conference of ministers of education begin discussing school closures to support infectious disease strategies (34). All education institutions were closed nationwide for about 2 months. Due to the federal system and state responsibility for education and health, each state implemented its own reopening strategy. Most reopened as early as 20 April 2020 for critical exams and transition grades (35) and fully reopened just after the 2020 summer holidays.

During school closures, children did not receive the benefits of school health promotion, including high-quality learning and educational opportunities, guidance from teachers and health professionals, access to digital infrastructure, meals, physical/sports activities, and social/cultural capital in terms of the school community and peer contact. As experience and evidence from other countries show (36) regarding schools during COVID-19 or past epidemics (37), adverse health effects are associated with closures, especially among socioeconomically disadvantaged children.

COVID-19 policies may have exacerbated pre-existing inequities, impacting healthy child development (32). Many children lack the necessary resources to ensure participation in distance learning, namely computers, internet access, and supporting adults. The education system and policy administration failed to systematically roll-out distance learning infrastructure, as teachers were not equipped to design, teach and support virtual learning.

### Lessons learned

COVID-19 impacted education institutions and fundamental interactions between school children and teachers (33,36,38). The German education system is not well prepared to respond to a pandemic, requiring a need to rethink and redesign the education system, to provide a more inclusive and health-literate system equipped to include health promotion. Working parents may not have the time and resources to support their children. With school closures, digital communication and learning has been the only option for education and school health promotion. Children therefore must be provided with the necessary digital infrastructure and skills to engage with this new form of virtual learning; all families should have capacities at their disposal, and teachers should receive the necessary training, including an emergency strategy for education. New educational approaches are needed, introducing more practical methods, adapted to real-world needs, and keeping health promotion activities effective (32,38).

## Case 4: policy focus — vulnerable groups-migrant workers (Singapore and Malaysia)

### The needs

Singapore and Malaysia are popular destinations for migrant workers from South and Southeast Asia. During the initial outbreak of COVID-19, while confirmed cases in Singapore were relatively low, the number of cases rose drastically in early April 2020, particularly among migrant workers (39). By mid-June, 39,223 cases (94.3%) of the total confirmed cases (41,615) in Singapore were migrant workers (40). Crowded dormitories and sharing of common facilities were identified as the root causes of the COVID-19 spread.

During the second week of March 2020, Malaysia became the worst COVID-19-hit country in Southeast Asia. The Malaysian government declared a Movement Control Order (MCO) on 18 March (41). The number of confirmed cases and community spread gradually decreased after the implementation of the MCO. In contrast, a marked escalation in new COVID-19 cases among migrant workers occurred since May 2020. By mid-June 2020, there were approximately 610 confirmed cases among documented migrant workers — 7.1% of 8535 cases in Malaysia (41).

### Immediate actions

The Singapore government quarantined 24 migrant worker dormitories, began COVID-19 testing, segregation of healthy and infected workers, and daily checks of fever/symptoms (39). Health authorities encouraged dormitory operators to maintain hygiene on their premises, non-governmental organizations (NGOs) provided meals, fruits and essential items, and temporary housing, such as vacant public housing flats, military camps, exhibition centers and floating hotels, was provided to enable physical distancing (39).

The Malaysian government enforced compulsory COVID-19 screening among migrant workers, financially covered by SOSCO (Social Security Organization) (42). NGOs collaborated with the government to provide food and essential supplies, and launched COVID-19 funds for marginalized groups, including migrant workers. The Malaysian Ministry of Health provides free testing and medical care for non-Malaysian citizens who have COVID-19 symptoms and their close contacts (41).

The Borderless Healthcare Group (BHG) implemented a total wellness program for migrant workers in several countries including Malaysia and Singapore. This online platform provides an opportunity for two million migrant workers to join the multi-lingual interactive COVID-19 education program and mental health support in their own language (43). In addition, the migrant workers from Bangladesh and Myanmar were allowed to consult doctors from their home countries free of charge via the online BHG platform (44).

### Long-term actions

The Singapore government announced that new dormitories with better facilities for migrant workers will be built to enhance physical distancing and hygiene practice (45). The Malaysian government also implemented a new law requiring employers to provide proper accommodation for the migrant workers to prevent the spread of COVID-19 (46).

### Lessons learned

Policies promoting health promotion messages on physical distancing, using personal protection equipment, particularly masks, and good hygiene were implemented during the COVID-19 outbreak. However, for migrant workers, poor living conditions and environment prevented them from practicing proper preventive measures. This is aligned with the recommendation of Lieberman *et al*. (47) to focus on policy and environmental changes rather than individually orientated health promotion programs. To support individual behavior change, it is critical to implement policies for enabling environments, supporting health literacy across all populations, especially those who are marginalized and most vulnerable (48).

## Case 5: cross-cutting SEM levels — intervention for the general population (Israel)

### The needs

Israel’s first reported case of COVID-19 was in February 2020, with a national lockdown declared in March 2020. As in-person health promotion programs were canceled to comply with stay-at-home guidelines (49), it became apparent that a national health promotion plan for emergency preparedness was lacking. Thus, health promotion experts from the national healthcare system set out to 1) learn about the needs of the public, including special populations, and 2) take decisions regarding appropriate action.

### The actions

The primary health promotion challenge focused on health literacy efforts to provide evidence-based information to the public, encouraging all to adopt behavioral measures for the individual and common good. The following topics received special attention during the outbreak, based on public interest: self-care in pregnancy/breastfeeding (50), smoking cessation, care for the elderly (51) and people with chronic disease, physical activity during home quarantine, and managing children’s media exposure. Language/cultural needs received attention, as information both printed and digital was provided in Hebrew and Arabic, and adjusted for the ultra-orthodox Jewish population. Additionally, at the peak of the first wave, the major religions in Israel celebrated major holidays (Passover, Ramadan and Easter). Recommendations promoted alternative ways in which these special holidays could be observed, without the ritual family gatherings, in order to maintain physical distancing. New health-promoting settings emerged, such as converted hotels for housing people with COVID-19 but who did not require hospitalization, where activities on dealing with stress, sexual health, healthy lifestyle, and more were implemented.

As digital healthcare solutions were rapidly developed during the pandemic, likewise safe and protected digital health promotion platforms were developed. Clalit Health Services, Israeli’s largest healthcare organization, allocated resources to digitally continue health promotion efforts. Virtual group workshops were launched on smoking cessation, healthy lifestyles for people with diabetes, nutrition/weight management for adults, birth preparation and breastfeeding. To date, following the second wave of the COVID-19 pandemic, over 600 virtual group sessions have been conducted, reaching over 800 people throughout the country.

A unique multidisciplinary research project, led by the Tel Aviv University Department of Health Promotion, in collaboration with the Collective IL Academia, produced a position paper entitled *TIME TO MOVE: urgent need to promote physical activity during the corona period*, emphasizing the devastating physical and mental consequences of physical activity restriction and advocating for promoting regular physical exercise. Recommendations for individuals, communities, media systems, and policy interventions were submitted to an Israeli parliament (Knesset) special committee convened in April 2020 to discuss the consequences of limiting physical activity, and whether to extend or lift restrictions on physical activities/sports (52).

### Lessons learned

Online consensus conferences were conducted throughout the outbreak among health promotion/public health professionals. Among the lessons learned and recommendations made were the need for a national and consistent voice during the outbreak, recognized and trusted by the public. Public participation was lacking in expressing the needs and recommending solutions for health promotion health literacy. The Collective IL Academia framework provided policy makers with reliable research for decision making in vital aspects of the pandemic, promoting transparency, trust, collaboration and public responsibility.

As Israel was confronted with a severe second wave of the pandemic, insights from the first wave were applied, yet still lacking is a national policy on health promotion preparedness for a health crisis, including health literacy interventions, public participation, and partnership.

## Discussion

These cases across diverse locations and health and education systems highlight challenges, recommendations, and solutions during the pandemic across various stages in the lifespan and levels of the SEM. We acknowledge that an individual-level focus only is not sufficient for sustainable behavior change, whereas a focus on interpersonal, organizational, community, policy or cross-cutting efforts are critical given the multilayered challenges in COVID-19. Struggles include coping with mental health, stress, loss of service accessibility, social connectivity and the needs of vulnerable populations. The cases provide researchers, practitioners and policy makers brief but rich examples highlighting multi-sectoral approaches across the levels of the SEM. Table 1 provides a summary of focal areas, health literacy needs identified, and lessons learned.

**Table 1.** Summary of cases across various dimensions.

Country	Life course focus	Area focus	SEM focus	Health literacy needs identified	Implications: research, practice and policy
Germany	Elders	Web of health/social needs	Interpersonal, including community and patient–provider relationships	Critical health literacy, including messages for solidarity/mutual support. Access to personal healthcare, including digital solutions.	Elders and other vulnerable populations can thrive during pandemics in supportive environments.
UK	Adults	Primary care/mental health	Organizational linked to policy to provide access to mental health advice and support	Mental health literacy and healthcare: understanding access to care options, reducing stigma for seeking care.	Primary care healthcare organizations are critical partners in improving access to mental healthcare and promoting mental health literacy.
Germany	Children	Schools	Community: school as hub for health-promoting relationships with families/children/teachers	Building holistic health literacy from school-based health promotion.	Highlights valuable role of schools for all children’s health needs; including digital solutions.
Singapore/Malaysia	Working-age adults	Migrant workers	Policies to mitigate COVID-19 with culturally-sensitive solutions addressing practical and social needs	Access to culturally relevant health information and structural support (food, safe shelter, access to in-language healthcare).	Environmental support for actions across all populations and culturally relevant resources.
Israel	Adult	General population	A national health promotion plan crossing SEM	Public health literacy, across language/cultural needs, offering digital solutions.	Underscores need for effective strategies to build public trust.

Key themes emerging from these cases directed especially at policy makers include:

- **Equity.** Addressing the needs of vulnerable populations in the appropriate language and cultural context is key. Efforts must meet people where they are, with what they need to stay healthy, while considering the inherent challenges of physical distancing. The needs of the hidden groups must be acknowledged, such as people with mental health challenges who refrain from seeking care, and children remaining at home, disconnected from technology whose family’s strife or hunger is often invisible.
- **Trust.** Transparency and clear communication are necessary to inform and build trust. This is vital during a pandemic, especially when its course is constantly changing, lingering longer than expected. Trust is built by acknowledging that guidelines reflect the best knowledge accrued to date, but may change as the science and study of the pandemic unfolds, especially to ensure patients’ understanding and meeting their needs. It is important to note that information collected through mass media and crowd sourcing may not be systematic nor accurate, and may amplify the detrimental infodemic.
- **Systems approach.** As seen in the cases presented, COVID-19 is not only an individual health issue but also engages caregivers, families, extended social networks, neighborhoods, communities, health systems and organizations, and governing/political systems. Structural changes in health promotion are requisite to sustain health during the COVID-19 and future pandemics.
- **Sustained action.** Existing health promotion policies during a crisis should not be ignored. A strong health promotion infrastructure must be maintained to meet both immediate and long-term needs. Ongoing programs such as routine vaccination, smoking prevention/cessation, and mammograms, must be sustained and resources must not be diverted elsewhere when addressing a health crisis such as COVID-19. The immediate and long-term effects of this pandemic on individual health, community well-being, and health systems are still emerging. While some nations remain in active crisis, nearly all are in a new normal with changes likely to stay, especially the shift to digital communication, interventions, and healthcare.

The current challenges include:

- building best practices and investing resources in health promotion focusing on health literacy, health education, accessible and appropriate health information, and healthcare, especially in a new technological age with social media and telehealth, during a pandemic;
- the need for valid and reliable data and evidence regarding health promotion/health literacy solutions for equitably improving and sustaining health for all, during an emergency;
- preparing for expected future challenges such as vaccine resistance and supporting those chronically disabled by COVID-19.

It is important to adopt community engagement strategies in the process of developing and maintaining relationships among the stakeholders to work together and promote well-being for long-term positive outcomes, based on trust, respect, and a sense of shared purpose. These relationships span all levels, from the micro (individual) to the meso (groups) to the macro (systems and institutions).

The pandemic is a call for humanity to remove inequities that divide, and engage in solidarity. Equity is the lighthouse to drive decision making, replacing survival of the fittest to face the pandemic with empowerment and support for people across the globe.
